# Liver Transplant Recipients Speak Out on Public Awareness and Education Surrounding Alcohol-Related Health Effects: A Survey Study

**DOI:** 10.7759/cureus.31760

**Published:** 2022-11-21

**Authors:** Shirley X Jiang, Katerina Schwab, Trana Hussaini, Mahmoud Omar, Ben Cox, Vladimir Marquez-Azalgara, Eric M Yoshida

**Affiliations:** 1 Medicine, University of British Columbia, Vancouver, CAN; 2 Pharmaceutical Sciences, University of British Columbia, Vancouver, CAN; 3 Gastroenterology, University of Alberta, Edmonton, CAN; 4 Hepatology and Gastroenterology, National Liver Institute, Menoufia University, Menoufia, EGY; 5 Gastroenterology, University of British Columbia, Vancouver, CAN

**Keywords:** liver transplant, survey study, public education, alcohol-related health effects, liver disease

## Abstract

Background: Compared to other recreational substances in Canada, alcohol consumption incurs the highest healthcare costs. Liver transplant recipients are unique stakeholders as members of the general public with lived experiences of liver disease. We sought to explore their perspectives on the current state of public education on alcohol-related health effects.

Methods: The most recent 400 liver transplant recipients at Vancouver General Hospital, Canada, were invited to participate in an anonymous online survey on alcohol-related health effects by mail, email, and phone.

Results: Of 372 contacted patients, 212 (57%) completed the survey. Most patients were between 60-79 years, 63% were male, and 69% were Caucasian. The most common liver conditions leading to transplant were viral hepatitis (33%), alcohol-related liver disease (16%), autoimmune liver disease (14%), and non-alcoholic fatty liver disease (15%). Most patients knew that alcohol leads to liver failure (85%), but fewer knew about alcohol leading to cancer (54%), heart disease (50%), and damage to other organs (58%). Most common sources of information included public media (61%), family and friends (52%), and physicians (49%), with narrative comments about learning of alcohol-related health effects after liver diagnosis. Most patients believed that public health education at a middle/high school level would have long-term efficacy (72%) compared to health warning labels (33%) and safety messaging in commercials (39%). Current public education was felt to be adequate by only 20% of patients and 73% of patients supported health warning labels.

Conclusions: Liver transplant patients reported a high, but not universal, awareness of alcohol-related health effects. A majority thought that current public health efforts were inadequate; it is critical to implement public health interventions to ensure consumers are able to make an informed decision on alcohol consumption.

## Introduction

In Canada, alcohol consumption leads to the highest socioeconomic and healthcare costs compared to other recreational substances but is associated with substantially lower public awareness of related health effects [1,2]. For example, while 94.8% of Canadian smokers were aware that smoking leads to lung cancer, only 25% of Canadians surveyed outside liquor stores knew that alcohol could lead to cancer [3,4]. Other health effects of alcohol include liver, heart, neuropsychiatric, and metabolic disease, with no established safe limit to consumption [5,6]. Despite these adverse outcomes, alcohol consumption continues to rise, partly due to the COVID-19 pandemic leading to social isolation and increased availability of alcohol from extended hours of sale, home delivery, and reduced minimum pricing in some provinces [7,8]. Thus, it is more important than ever to ensure that consumers are able to make informed decisions surrounding alcohol consumption.

 Providing consumers with warning statements on the chronic conditions caused by alcohol has been shown to decrease consumption intentions [9]. Public education through health warning labels (HWLs), counter-advertising, school-based education initiatives, and work-based programs have all demonstrated positive effects in the literature [10-14]. Despite these available methods of public education, the Canadian Alcohol Policy Evaluation project recently found that Health and Safety Messaging was the least implemented policy domain at only 25.7% across the country [15].

 Liver transplant patients are uniquely poised as stakeholders in evaluating public awareness of alcohol and public health interventions as they are members of the general public with a considerable understanding of liver disease from their own lived experiences. As emphasized by a recent Canadian Medical Association Journal editorial, it is integral to engage the appropriate patient experts in patient-centred research and health policy [16]. No literature currently lends a voice to liver transplant patients to comment on alcohol awareness and existing public health interventions.

This article was previously presented as a meeting abstract at the 2022 Liver Meeting of the American Association for the Study of Liver Disease on November 6, 2022.

## Materials and methods

Study design

We conducted an anonymous online survey on awareness of alcohol-related health effects in post-liver transplant patients followed at Vancouver General Hospital, the liver transplant centre of British Columbia, Canada. The study population was comprised of 400 patients who received a liver transplant for any aetiology, who were older than 18 years by March 2022, and who had current phone and/or mailing addresses. Patients were invited to participate in the survey once by mail and up to two times by phone; if patients wished to complete the survey verbally by phone, this was conducted by the research team after obtaining consent. This study was approved by the University of British Columbia Clinical Research Ethics Board.

Survey tool

The survey was hosted on the Qualtrics platform and took an average of 6.5 minutes to complete. Consent was obtained verbally by phone or online prior to the survey. The survey consisted of 17 items surrounding patients’ clinicodemographic information, personal experiences with public education on the health effects of alcohol (including in comparison to public education on smoking cigarettes), and perceived efficacy of public health interventions for different durations (days, months, and years). The survey was open from February 11 to May 15, 2022.

Data analysis

Descriptive statistical data analysis was conducted. Participant responses were described as counts and proportions based on the total number of responses for each question. Descriptive fields were available for narrative comments about alcohol awareness and public education.

## Results

Demographic information

Of the 400 most recent patients to receive a liver transplant, 372 patients had current contact information. Of these, 212 patients (57%) completed the survey (Table 1). Most patients (124, 62%) were 60-79 years old, 55 patients (27%) were 40-59 years old, and 16 patients (8%) 20-39 years old. Almost two-thirds (63%) of patients were male. Most patients were Caucasian (69%), East Asian (17%), and South Asian (5%). Annual household income was >$100,000 in 32% of patients, $50-100,000 in 29% of patients, $20-50,000 in 28% of patients, and less than $20,000 in 12% of patients. Most patients had either a high school degree (23%), a university or college degree (42%), or a graduate/professional degree (18%). Self-reported liver conditions included viral hepatitis in 67 patients (33%), autoimmune liver disease (AILD) in 29 patients (14%), ALD) in 32 patients (16%), and non-alcoholic fatty liver disease (NAFLD) in 30 patients (15%).

**Table 1:** Clinicodemographic information of survey respondents *Calculated based on total number of responses per question. +Includes polycystic liver disease (n=4), biliary atresia (n=3), hereditary hemochromatosis (n=3), alpha-1 antitrypsin deficiency (n=2), genetic syndrome NOS (n=2), polycythemia rubra vera (n=1), and Wilson’s disease (n=1).

Variable	Number (%)*
Total respondents	212
Age	
<20 years	1 (0.5%)
20-39 years	16 (8%)
40-59 years	55 (27%)
60-79 years	124 (62%)
>80 years	5 (3%)
Male	121 (63%)
Ethnicity	
Caucasian	135 (69%)
East Asian	34 (17%)
Hispanic	3 (2%)
South Asian	9 (5%)
Other	13 (7%)
Annual household Income	
< $20,000	22 (12%)
$20-50,000	52 (28%)
$50-100,000	55 (29%)
>$100,000	60 (32%)
Highest level of education completed	
Middle school degree	8 (4%)
High school degree	46 (23%)
Vocational degree	23 (12%)
University or College degree	84 (42%)
Masters, PhD, or Professional Degree	35 (18%)
Liver condition	
Autoimmune hepatitis	29 (14%)
Alcohol-related liver disease	32 (16%)
Non-alcoholic fatty liver disease	30 (15%)
Viral hepatitis	67 (33%)
Primary sclerosing cholangitis	21 (10%)
Primary biliary cholangitis	6 (3%)
Drug induced liver injury	6 (3%)
Hepatocellular carcinoma	6 (3%)
Unknown	14 (7%)
Other+	18 (8%)

Experiences of public education

**Figure 1:**
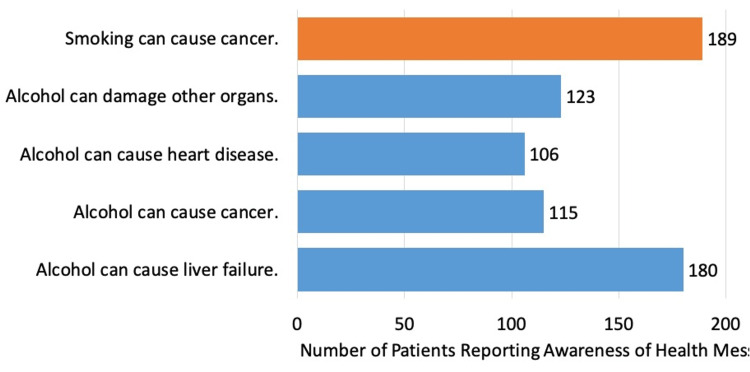
Public education on health-related effects of alcohol compared to other health messaging

Most patients (85%) reported being taught that alcohol can lead to liver failure, compared to 89% who were taught that smoking cigarettes can cause cancer. Only slightly more than half of patients reported being taught other health-related effects of alcohol; 115 patients (54%) were aware that alcohol can lead to cancer, 106 patients (50%) knew of the association with heart disease, and 123 (58%) were aware of damage to other organs (Figure 1).

**Figure 2:**
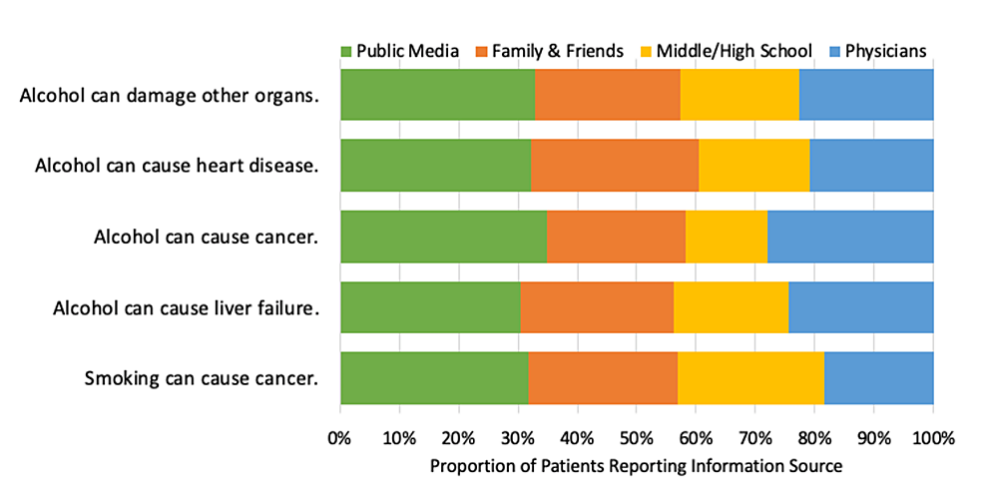
Sources of information for each health message* *As a proportion of all reported sources of information for each health message

The most common reported sources of information for alcohol causing liver failure were public media (61%), family and friends (52%), physicians (49%), and, least of all, middle/high school (39%). Comparatively, education on smoking cigarettes originated from public media (67%), family and friends (53%), middle/high school (52%), and, much less, physicians (39%). Education on alcohol leading to cancer originated from public media (51%), physicians (41%), family and friends (34%), and middle and high school (20%). Patients were taught about alcohol leading to heart disease by public media (48%), family and friends (42%), physicians (31%), and middle and high school (28%). Finally, education on alcohol damaging other organs was provided by public media (54%), family and friends (40%), physicians (37%), and middle and high school (33%) (Table 2, Figure 2).

**Table 2:** Public education on health-related effects of alcohol and information sources

Teaching	Number (% total patients) Taught	Information Sources, N (%)			
		Middle/High School	Public Media	Family & Friends	Physicians
Alcohol can cause liver failure.	180 (85%)	70 (39%)	110 (61%)	94 (52%)	88 (49%)
Alcohol can cause cancer, including liver cancer.	115 (54%)	23 (20%)	59 (51%)	39 (34%)	47 (41%)
Alcohol can cause heart disease.	106 (50%)	30 (28%)	51 (48%)	45 (42%)	33 (31%)
Alcohol can damage other organs.	123 (58%)	40 (33%)	66 (54%)	49 (40%)	45 (37%)
Smoking can cause cancer.	189 (89%)	99 (52%)	127 (67%)	101 (53%)	74 (39%)

There were several narrative comments on self-education, learning from personal experiences, limited public health interventions, and learning about alcohol-related health effects only after contact with the medical system for liver disease (Table 3).

**Table 3:** Comments on sources of education on alcohol-related health effects

Theme	Example comments
Self-education	“Self-research”, “personal research online”, “Internet”, “reading up on liver disease”, “own research at library”, “reading articles”
Personal experiences	“friends with cirrhosis”, “father had liver disease with heart problem”
Diagnosis at medical contact	“first heard when diagnosed with cirrhosis”, “when getting transplant”, “had not heard before my doctor”, “learned after was diagnosed with hepatitis”, “solid organ transplant clinic”
Critiques on public education	“in the media I heard more about smoking than alcohol”, “Information about alcohol was prevalent, information to help understand addiction was not”
Public health interventions	“commercials on TV”, “heard about the same [things] about alcohol and smoking”, “labels on beer cans”

Opinions on public health interventions

**Figure 3:**
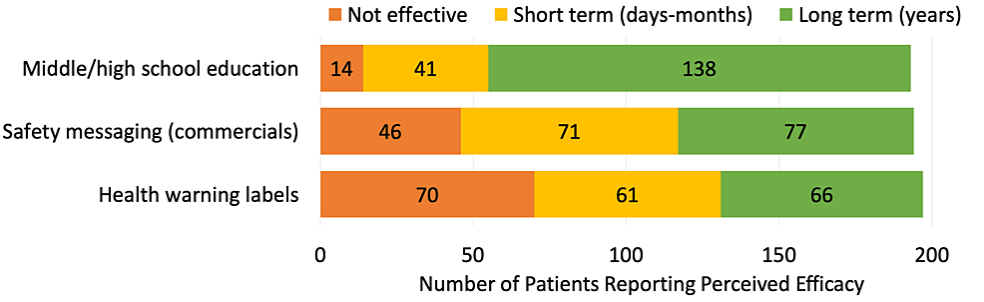
Perceived efficacy of public health interventions

Forty patients (20%) felt there was adequate general public education on the health effects of alcohol. Most patients believed that education on alcohol-related health effects at a middle or high school level would be most effective in the long term at 72%. Only 14 patients (7%) felt that school-based education would be ineffective, compared to 70 patients (36%) and 46 patients (24%) who felt HWLs and safety messaging, respectively, would be ineffective. Use of health warning labels (HWLs) and safety messaging in commercials were believed to have lower effectiveness in the short-term and long term at 31% and 33% for HWLs, respectively, and 37% and 39% for safety messaging, respectively (Table 4, Figure 3). Most patients (145, 73%) supported government-mandated health warning labels.

**Table 4:** Perceived efficacy of public health interventions

Intervention	Perceived Efficacy		
	Not effective	Short-term (days-months)	Long term (years)
Health warning labels	70 (36%)	61 (31%)	66 (33%)
Safety messaging (commercials)	46 (24%)	71 (37%)	77 (39%)
Middle/high school education	14 (7%)	41 (21%)	138 (72%)

Patients with alcohol-related liver disease (ALD)

Of 30 patients reporting ALD, 21 (68%) were aged 60-79, 25 (83%) were male, 25 (83%) were Caucasian, and 21 (70%) had a post-secondary degree. Almost half (47%) of patients also endorsed an additional liver etiology including non-alcoholic fatty liver disease (five patients), viral hepatitis (five patients), and autoimmune liver disease (three patients). Patients with ALD reported slightly higher awareness of alcohol causing liver failure (90%); but similar to the general group, fewer were taught that alcohol can lead to malignancy (47%), heart disease (43%), and damage other organs (60%). Reported sources of education were also predominantly public media, family and friends, and physicians. Compared to the general group, only 14 patients (50%) with ALD believed that education at a middle or high school level would have long-term effectiveness. The expected long-term efficacy of HWLs and safety messaging was similar to the general group at 31% and 41%, respectively. Similarly, 20% of patients with ALD believed there was sufficient public education on alcohol-related health effects and 73% endorsed government-mandated HWLs.

## Discussion

Our study is the first to report on public education surrounding alcohol consumption through the perspectives of liver transplant patients, who have personal experience with both public health education and liver failure. Liver transplant patients reported a high (85%) but not universal knowledge of alcohol leading to liver failure. The most commonly reported sources of education on alcohol were public media, family and friends, and physicians. It was concerning that several narrative comments reported first learning about alcohol-related health effects upon diagnosis of liver disease, representing missed opportunities for public education outside of a transplant setting. Comparatively, more participants (89%) reported learning of the hazards of smoking cigarettes, an issue that enjoys mandated public health interventions including health warning labels, public media coverage, and integration into the school curriculum. Accordingly, physician counselling was the least reported information source on cigarettes, showing that high levels of awareness can and should be achieved with a robust public health strategy.

When considering specific public education points, awareness of alcohol leading to cancer remained a crucial but understated fact. In a previous study of alcohol consumers in Yukon, Canada, only 25% reported being aware that alcohol can lead to cancer [4]. In our study, 54% of liver transplant patients reported being aware that alcohol causes cancer, however, this is still lower than expected considering the counselling provided to liver transplant patients. It is crucial to emphasize the link between alcohol and cancer as improving knowledge has been found to increase consumer support for alcohol public health policies [17-19]. Additional public education specifically on alcohol as a carcinogen is required and can synergistically provide momentum for public health interventions.

Only a minority of liver transplant patients (20%) felt that public education on alcohol-related health effects was adequate. More patients endorsed school-based programs (93%) over safety messaging (76%) and HWLs (64%). There is literature to support all three methods of public education to increase awareness but, in keeping with liver transplant recipient perceptions, behaviour change is less predictable for safety messaging and HWLs. A meta-analysis of randomized studies on school-based programs found a reduction in alcohol use in adolescents though there was no impact detected on lifetime alcohol use and some studies found a “boomerang” effect, whereby alcohol use increased in the interventional group [13]. Studies on alcohol warning commercials have all noted increased awareness and, in some studies, there was a corresponding reduction in alcohol consumption; however, this was not uniform [12,20,21]. It is possible that this is due to the short duration of exposure to warning campaigns in the face of extensive alcohol advertising. Finally, while HWLs have changed health behaviours in some studies, their impact is conflicting in literature and likely depends on effective label design [10,11,22]. Nonetheless, the literature is in agreement that HWLs increased awareness of alcohol-related health effects, and thus, HWLs are recommended by the World Health Organization as a cost-effective tool to directly target consumers at key points of sale and use, with the highest exposures to heavy drinkers [23]. HWLs are also well supported by consumer focus groups and the majority of respondents (73%) in our survey [24]. Given that advertisements for alcohol are abundant and safety messaging is relatively rare across Canada [25], increasing public education through evidence-based methods is needed to enable consumers to make informed decisions on alcohol consumption.

There are several limitations to our study. As we used an online survey design, liver transplant recipients who do not use technology may be dissuaded from responding and represent a group with different information sources; to mitigate these effects, all patients were also contacted by mail and phone with the option to complete the survey verbally. As well, while our response rate of 57% is comparable to a previous study conducted on the same population at 42%, there is still a significant proportion of responses not captured [26]. There may be a selection bias as patients with strong opinions on the topic are more likely to respond, particularly those with alcoholic liver disease due to their personal experiences. Compared to a retrospective Canadian study examining aetiologies leading to liver transplantation, our respondents reported a higher rate of NAFLD and a lower rate of hepatocellular carcinoma, but the representation of other liver aetiologies was similar, including ALD [27]. Further, responses of patients with ALD were analysed in comparison to the general study population and found to be largely similar. As well, our respondents were predominantly Caucasian, which may not represent the experiences of other ethnic minorities, particularly as a previous study of British Columbian patients referred for liver transplantation found significantly higher rates of ALD in South Asians, signalling higher alcohol use [28]. Our sample size was unfortunately too limited to conduct subgroup analysis stratified by other clinicodemographic factors such as age, education level, and income, though these are associated with health literacy and health outcomes. Future studies on awareness of alcohol-related health effects that are able to examine these factors would be better positioned to target public health interventions to specific groups. Finally, as the initial study of liver transplant patient experiences, we only included conventional methods of public education; further study should include social media and phone application-based initiatives, particularly in younger populations [29,30].

## Conclusions

Alcohol consumption, with its associated morbidity and mortality, is growing in Canada but health and safety messaging remain limited. Liver transplant patients had a high awareness of alcohol leading to liver failure but less knowledge of cancer risk and other related health effects, demonstrating the need for more public education even in this highly medicalized population. As unique stakeholders of the public with lived experience of liver disease, the majority of liver transplant patients felt that current public health efforts were inadequate and supported school-based programs, safety messaging, and government-mandated health warning labels. Public health interventions are sorely needed to increase awareness of alcohol-related health effects so that consumers are able to make informed decisions surrounding alcohol.
